# Measurement of outflow resistance imposed by magnetic sphincter augmentation: defining normal values and clinical implication

**DOI:** 10.1007/s00464-020-08068-4

**Published:** 2020-10-13

**Authors:** Shahin Ayazi, Andrew D. Grubic, Ping Zheng, Ali H. Zaidi, Katrin Schwameis, Adam C. Alleyne, Brittney M. Myers, Ashten N. Omstead, Blair A. Jobe

**Affiliations:** Esophageal Institute, Allegheny Health Network, 4815 Liberty Avenue, Suite 439, Pittsburgh, PA 15224 USA

**Keywords:** Magnetic sphincter augmentation (MSA), High-resolution manometry, Gastroesophageal junction, Dysphagia, Intrabolus pressure (iBP)

## Abstract

**Introduction:**

No manometric criteria have been defined to select patients for magnetic sphincter augmentation (MSA). The first step to establish such criteria is to measure the outflow resistance at esophagogastric junction (EGJ) imposed by MSA. This resistance needs to be overcome by the esophageal contraction in order for the esophagus to empty and to avoid postoperative dysphagia. This study was designed to measure the outflow resistance caused by MSA in patients free of postoperative dysphagia.

**Methods:**

Records of the patients who underwent MSA in our institution were reviewed. A group of MSA patients with excellent functional outcome, who were free of clinically significant postoperative dysphagia, were selected. These patients then underwent high-resolution impedance manometry (HRIM) at a target date of 1 year after surgery. The outflow resistance was measured by the esophageal intrabolus pressure (iBP) recorded 2 cm proximal to the lower esophageal sphincter (LES).

**Results:**

The study population consisted of 43 patients. HRIM was performed at mean of 20.4 (10.4) months after surgery. The mean (SD) amplitude of the iBP was 13.5 (4.3) before surgery and increased to 19.1 (5.6) after MSA (*p* < 0.0001). Patients with a smaller size LINX device (≤ 14 beads) had a similar iBP when compared to those with a larger device (> 15 beads) [19.7 (4.5) vs. 18.4 (5.9), *p* = 0.35]. There was a significant correlation between the iBP and % incomplete bolus clearance [Spearman *R*: 0.44 (95% CI 0.15–0.66), *p* = 0.0032]. The 95th percentile value for iBP after MSA was 30.4 mmHg.

**Conclusion:**

The EGJ outflow resistance measured by iBP is increased after MSA. The upper limit of normal for iBP is 30 mmHg in this cohort of patients who were free of dysphagia after MSA. This degree of resistance needs to be overcome by distal esophageal contraction and will likely be requisite to prevent persistent postoperative dysphagia.

Magnetic sphincter augmentation (MSA) is a safe and effective surgical treatment for patients with gastroesophageal reflux disease (GERD). This procedure is a technically straightforward and highly reproducible outpatient procedure, and multiple centers across the United States and Europe have reported a high degree of success with consistent clinical outcomes [1–3]. MSA is also considered to be a less invasive surgical option compared to Nissen fundoplication as it preserves gastric anatomy and is reversible [4, 5].

MSA has been applied with the increasing frequency in the treatment of patients with reflux disease and it is currently offered in more than 300 centers across the US. Despite such a broad adoption, no manometric criteria have been defined to aid in patient selections for this procedure.

The LINX device applies magnetic force to augment the barrier function of an incompetent lower esophageal sphincter (LES). This augmentation will result in increased resistance to flow at the esophagogastric junction (EGJ) during a peristaltic contraction. The first step to establish manometric criteria to aid in patient selection is to measure the outflow resistance at the EGJ imposed by MSA in patients with no postoperative dysphagia. The intrabolus pressure (iBP) is an indicator of outflow resistance caused by the LES. Studies have applied this parameter to measure outflow resistance caused by a Nissen fundoplication and this has been linked to outcome [6, 7].

The goals of this study were to compare the EGJ outflow resistance before and after magnetic augmentation in a group of patients with no clinically significant postoperative dysphagia and excellent functional outcome. We also aimed to calculate the upper limit of outflow resistance in this cohort of patients. This degree of resistance needs to be overcome by distal esophageal smooth muscle contraction and will likely be requisite to prevent persistent postoperative dysphagia.

## Methods

Records of the patients who underwent MSA at Allegheny Health Network hospitals (Pittsburgh, PA) were reviewed. A group of patients with excellent functional outcome, who were free of clinically significant postoperative dysphagia, were approached for objective foregut testing. Approval was obtained from the Allegheny Health Network institutional review board (IRB 2018-161) prior to the start of the study.

Patients were 18 years or older with persistent GERD or laryngopharyngeal reflux (LPR) symptoms despite maximal anti-secretory therapy. All patients had objective evidence of reflux disease based on increased esophageal acid exposure on pH monitoring or a positive impedance–pH based on previously described criteria [8, 9]. None of the patients had a previous history of esophageal or gastric surgery, gross anatomic abnormalities, such as esophageal stricture, significant esophageal dysmotility, or a known allergy to titanium.

### Preoperative assessment

All patients completed a detailed clinical evaluation with a focus on their foregut symptoms and acid suppression medication use and completed the gastroesophageal reflux disease-health-related quality of life (GERD-HRQL) and reflux severity index (RSI) questionnaires while taking their usual dosing of anti-secretory medication. The GERD-HRQL assesses GERD symptoms and patient satisfaction using a 0–5 rating scale. It is composed of ten questions relating to the severity of heartburn, regurgitation dysphagia, odynophagia, and bloating [10]. The total GERD-HRQL score is calculated by summing the responses to the 10 questions with scores ranging from 0 to 50 [11]. Similarly, the RSI is a validated questionnaire used in assessment of LPR symptoms. It consists of 9 items with each scored using a 0–5 rating scale, with total score ranging from 0 to 45 [12].

Patients also completed an objective foregut evaluation prior to consideration for surgery. This evaluation consisted of the following tests:

1. Esophagogastroduodenoscopy (EGD) with biopsy: to assess the presence of esophagitis, Barrett’s esophagus, and the presence and size of a hiatal hernia.
2. High-resolution impedance manometry (HRIM): This test was performed in a similar fashion in all patients before and after MSA. Following an overnight fast, a 36-channel catheter with circumferential sensors 1 cm apart along the catheter (Medtronic Inc., MN) was passed through nasal channel and advanced such that three or more recording ports had an intragastric location. Patients were then asked to swallow 5 ml of room temperature normal saline spaced at 20- to 30-s intervals for 10 swallows in the Fowler’s position. Studies were then analyzed using dedicated HRM analysis software (ManoView; Medtronic Inc., MN) [13].
3. Esophageal pH or impedance-pH monitoring: These tests were performed selectively using either Bravo pH monitoring (Medtronics, Shoreview, MN, USA) or multichannel intraluminal impedance (MII) pH monitoring (Sandhill Scientific Inc, Highlands Ranch CO). Prior to pH testing proton pump inhibitors were discontinued for 10 days. A DeMeester score > 14.7 was considered as abnormal distal esophageal acid exposure. Impedance–pH testing was used in patients with predominate symptoms of LPR with or without typical reflux symptoms using previously described criteria [8, 9].
4. Videoesophagram: This imaging study was done to evaluate gross pharyngeal and esophageal motility; to further delineate the anatomy and assess for any potential mass or mucosal lesions, diverticulum; and to evaluate hiatal hernia and esophageal stricture or scarring.

### Implant and surgical procedure

The LINX device (Ethicon, Johnson & Johnson, Shoreview, MN) consists of a series of titanium beads with magnetic cores hermetically sealed inside. The beads are interlinked with independent titanium wires to form a flexible and expandable ring with a “Roman arch” configuration. Each bead can move independently of the adjacent beads, creating a dynamic implant without limiting esophageal range of motion. The device is manufactured in different sizes, ranging from 13 to 17 beads, and is capable of nearly doubling its diameter when all beads are separated.

MSA is performed laparoscopically and consists of complete posterior mediastinal esophageal mobilization with restoration of intra-abdominal esophageal length (≥ 3 cm), interrupted posterior crural closure (without pledgets or mesh) and device placement at the level of the EGJ with the posterior vagus nerve trunk located on the outside of the magnetic ring. A sizing procedure, which assesses esophageal circumference, is performed prior to selecting the size of the device.

### Postoperative and outcome assessment

In our practice, all patients undergo routine postoperative visits at 2 weeks, 6 weeks, 6 months, and then yearly after surgery. During these visits they are assessed for resolution of their reflux symptoms, use of anti-secretory medication, and procedure-related complications. They complete the GERD-HRQL and RSI questionnaires at their 6 months and yearly visits.

### Final study population

A group of patients found to have excellent functional outcome defined by resolution of the primary presenting reflux symptom, who were free of clinically significant dysphagia, were considered for this study. They were approached for objective testing at a minimum of 1 year after their surgery. Patients underwent upper endoscopy to confirm appropriate position of the LINX device. Clinically significant dysphagia was defined as a postoperative dysphagia score > 3 on the “difficulty swallowing” item of the GERD-HRQL questionnaire. Objective testing consisted of the same tests employed in the preoperative evaluation.

### Analysis of manometry studies and measurement of outflow resistance

Manometry studies were analyzed using the latest versions of ManoView analysis software (V 3.0.1 and V 3.3). Standard manometric parameters included LES overall and abdominal lengths and resting pressure, integrated relaxation pressure (IRP), and distal contractile integral (DCI). The iBP was measured for each swallow 2 cm proximal to the LES during the emptying phase of esophageal peristaltic topography, using software tools embedded in the older version of the analysis software. This metric measures the mean of the maximum pressure within the designated window (starting at the upper esophageal sphincter relaxation and ending at the contraction front) over a 3 s width relative to gastric pressure. The mean of the iBP measurements across ten swallows constituted the mean iBP for each patient.

### Statistical analysis

Values are expressed as either mean with standard deviation (SD) or median with interquartile range (IQR). Postoperative outcome variables and manometric measures, including iBP, were compared to the preoperative values using nonparametric Mann–Whitney *U*-test, Wilcoxon signed-rank test, and Pearson’s Chi-square test when appropriate. The correlation between postoperative iBP values and relevant preoperative and postoperative measures were performed using Spearman test and expressed as the correlation coefficient *R* with 95% confidence intervals (CI). The upper limit of normal was defined using the 95th percentile value. A *p* value < 0.05 was considered statistically significant. Statistical analysis was performed using SAS software (SAS Institute Inc., Cary, N.C.).

## Results

The study population consisted of 43 patients. The demographic and baseline clinical characteristics of these patients are shown in Table 1. At a mean follow-up of 22.7 (13.1) months, all patients had resolution of their primary presenting reflux symptom and were free of clinically significant dysphagia. GERD-HRQL and RSI total scores were significantly improved after surgery (Table 2). Freedom from use of anti-secretory medications was reported by 92% of the patients and normalization of the distal esophageal acid exposure was found in 77% of patients.

**Table 1:** Demographic and baseline clinical characteristics of the study population

Characteristic	*N* (%)
Age (year)	
Mean (SD)	54.0 (14.6)
Gender	
Male (%)	15 (34.9%)
Female (%)	28 (65.1%)
BMI	
Mean (SD)	28.9 (4.6)
DeMeester score	
Mean (SD)	33.9 (32.4)
Presence and size of hiatal	
No hernia	11 (25.6%)
Small (≤ 3 cm)	20 (46.5%)
Large (> 3 cm)	10 (23.2%)
Paraesophageal hernia	2 (4.7%)

**Table 2:** Comparison of quality of life measures before and after MSA

Measures	Preoperative (Mean, SD)	Postoperative (Mean, SD)	*p* value
GERD-HRQL total score	33.3 (18.6)	9.6 (12.0)	< 0.001
RSI total score	22.8 (10.5)	11.2 (9.5)	< 0.001

Magnetic augmentation of the sphincter resulted in a significantly higher LES abdominal length and higher residual pressure (Table 3). When compared to baseline values, there was also an increase in the LES overall length and resting pressure, but these differences did not reach the statistical significance (Table 3).

**Table 3:** Comparison of the manometric characteristics of the LES and esophageal body before and after surgery

	Preoperative Mean (SD)	Postoperative Mean (SD)	*p* value
LES overall length, cm	3.0 (0.6)	3.3 (0.8)	0.093
LES abdominal length, cm	0.9 (0.9)	1.4 (1.0)	0.041
LES resting pressure, mmHg	23.3 (12.7)	26.8 (16.8)	0.203
LES residual pressure, mmHg	8.4 (4.3)	12.7 (7.3)	0.002
DCI, mmHg.s.cm	2327 (2630.9)	2758.8 (2608.7)	0.022
% peristalsis	88.7 (19.5)	86.4 (18.6)	0.304
% incomplete bolus clearance	20.5 (31.9)	20.6 (32.5)	0.930

**Fig. 1:**
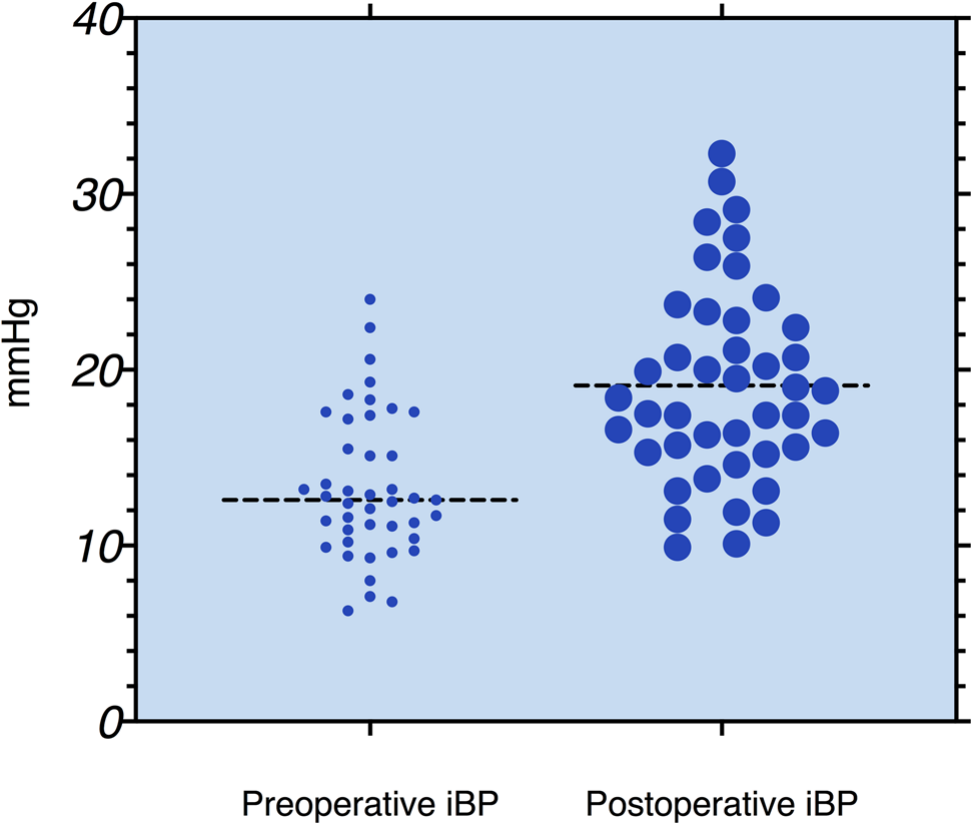
Comparison of preoperative and postoperative iBP values showing significant increase in the iBP after MSA (*p* < 0.001, Wilcoxon matched pair test)

**Fig. 2:**
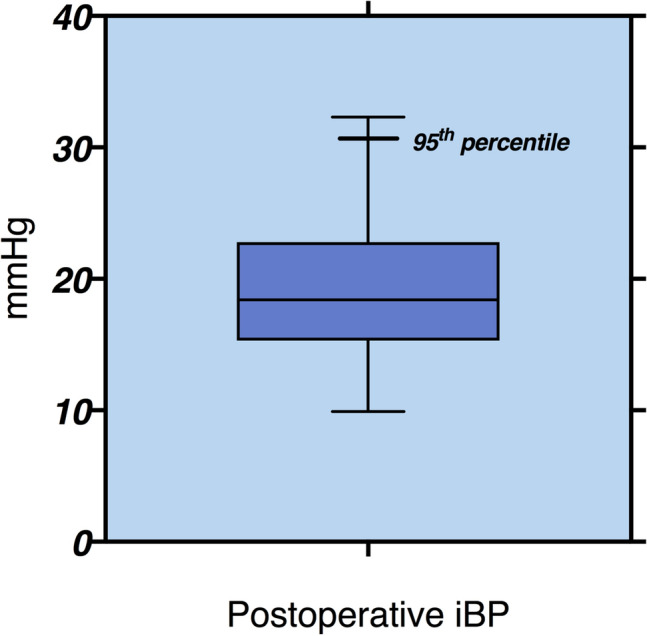
The iBP value after MSA are presented as box (median, 25th, and 75th percentiles) and whisker (minimum and maximum) plots. The 95th percentile value (upper limit of normal) for iBP after MSA in patients with no dysphagia is 30.4 mmHg

The mean (SD) amplitude of iBP was 13.3 (4.1) mmHg before surgery and increased to 19.2 (5.6) mmHg after MSA (*p* < 0.001, Fig. 1). The 95th percentile value for iBP after MSA was 30.4 mmHg (Fig. 2). The median size of the LINX device used was a 14 bead system (IQR: 14–15). Patients with a smaller-sized LINX device (≤ 14 magnetic beads) had a similar iBP when compared to those with a larger device (> 15 magnetic beads) [19.7 (4.5) vs. 18.4 (5.9), *p* = 0.35].

Postoperative iBP did not correlate with the resting pressure of the magnetically augmented LES (*p* = 0.31), but there was a correlation between iBP and IRP after MSA [Spearman *R*: 0.42 (95% CI 0.13–0.64, *p* = 0.0053]. DCI increased after MSA [2327 (2630.9) mmHg·s·cm vs. 2758.8 (2608.7) mmHg·s·cm, *p* = 0.022], but there were no changes in the percentages of incomplete bolus clearance [20.5 (31.9) vs. 20.6 (32.5), *p* = 0.93] and intact primary peristalsis [88.7 (19.5) vs. 86.4 (18.6), *p* = 0.30] when compared with baseline values.

**Fig. 3:**
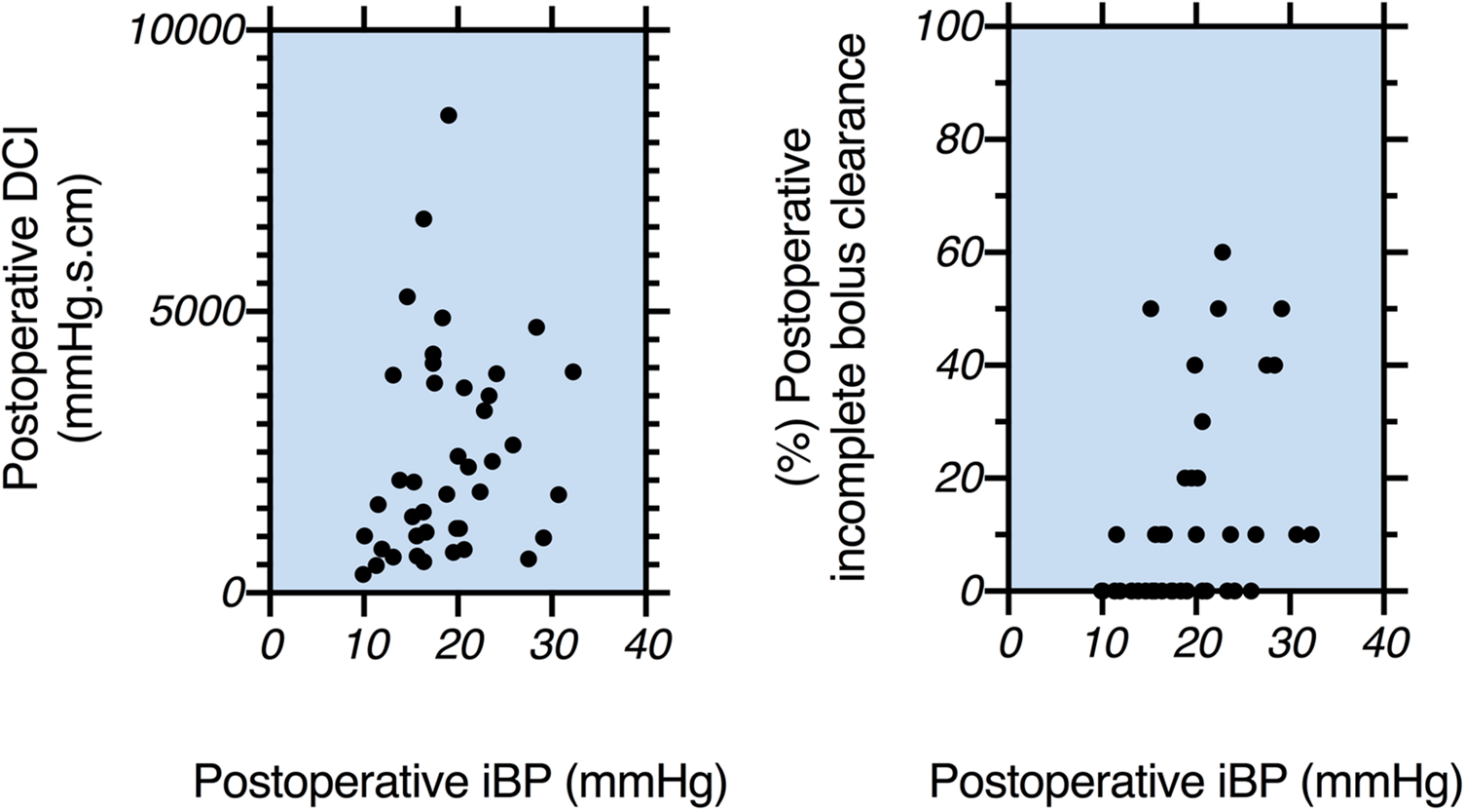
The iBP measurement after MSA is directly correlated with DCI and % incomplete bolus clearance. The Spearman R correlation coefficient values were as follows: 0.31 (95% CI 0.03–0.57), *p* = 0.042 and 0.44 (95% CI 0.15–0.66), *p* = 0.0032, respectively

The iBP after MSA correlated directly with DCI [Spearman *R*: 0.31 (95% CI 0.03–0.57), *p* = 0.042] and the percentage of incomplete bolus clearance [Spearman *R*: 0.44 (95% CI 0.15–0.66), *p* = 0.0032] as shown in Fig. 3. No correlation was found between iBP and failed peristalsis (*p* = 0.16).

Resting LES pressure was the only preoperative manometric parameter with a correlation to postoperative iBP [Spearman *R*: 0.73 (95% CI 0.63–0.80, *p* < 0.0001].

## Discussion

**Fig. 4:**
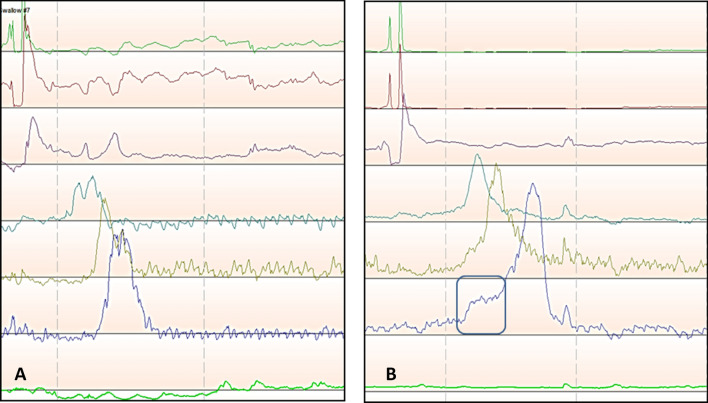
**A** HRM line tracing of a GERD patient prior to MSA showing a low iBP, **B** HRM line tracing of the same patient after MSA with elevated iBP (black rectangle), recognized by rise in pressure to a plateau above the esophageal baseline just preceding the upstroke of esophageal contraction wave

It is critical to identify the physiologic changes which occur following MSA in order to guide patient care. In this study, we investigated the resistance imposed by MSA in a group of patients who were free of dysphagia at a median of 20.4 months after surgery. The major finding of this study is that the EGJ outflow resistance measured by iBP is increased from baseline values after MSA (Fig. 4). The upper limit of normal for iBP in this cohort was 30 mmHg, compared to the 20 mmHg value reported for Nissen fundoplication [6]. The higher values for iBP after MSA supports prior notions that the LINX device imposes more resistance at the EGJ compared to Nissen fundoplication, and this consequence likely reflects the higher incidence of persistent postoperative dysphagia associated with MSA [14]. Therefore, the implication is that a given force of esophageal peristalsis is required to overcome the elevated resistance imposed by the LINX device and that this factor needs to be incorporated into patient selection.

Previous studies have investigated the impact of MSA on LES barrier function. These studies are of value when attempting to understand the requisite baseline esophageal function required for successful outcome with MSA. Warren and colleagues showed that MSA restored the manometric competency of the LES in 77% of patients with a defective LES [15]. This same study noted significant increases in LES resting pressure, residual pressure, and length. Fifteen percent of the study patients reported dysphagia; however, LES residual pressure (7.5 vs. 7.4 mmHg) and percentage of peristalsis (95% vs. 100%) after MSA was similar between patients with or without dysphagia. Interestingly patients with dysphagia were noted to have higher distal esophageal amplitude (93.1 vs. 82.8 mmHg) [15]. Similarly, Riva et al. reported comparable LES findings with increases in IRP and LES length, with 49% of patients in this study experiencing dysphagia; however, IRP was not different between patients with and without dysphagia [16].

The focus of the recent studies has been on the LES resting pressure and IRP as the markers of resistance at the EGJ, and IRP is a major component of the Chicago Classification in the characterization of several disorders [17, 18]. While this emphasis allows for simplicity and standardization, it does not take into account all factors particularly as related to surgical therapies. Chicago classification was designed and intended for patients without prior surgery. In this classification, the IRP value > 15 mmHg is used to define EGJ outflow obstruction (EGJOO) [19]. The median (IQR) of the IRP in our study population was 12.2 (9.5–14.4). Nine patients (21%) in our cohort would be defined as having EGJOO using cutoff value of 15 mmHg. None of these patients had symptoms suggestive for EGJOO and had preserved peristaltic function and bolus clearance. This observation highlights the need for development of novel manometric measures that are applicable to the surgical population.

Intrabolus pressure (iBP) is a manometric measurement of the force exerted on a bolus during esophageal transit. It is a complex measurement which takes into account not only contractile force but also resistance. The concept was first used for pharyngeal swallowing through the upper esophageal sphincter but was subsequently applied to the LES [20, 21]. Four phases of esophageal swallowing have been described: (I) esophageal accommodation, (II) compartmentalization, (III) esophageal emptying, and (IV) ampullary emptying [22]. During transition from phase II to phase III esophageal peristalsis generates contractile force against the resistance of the LES. The pressure gradient just prior to contraction during this transition is the iBP. Once peristaltic force is greater than the resistance, a bolus will begin transit through the EGJ.

**Fig. 5:**
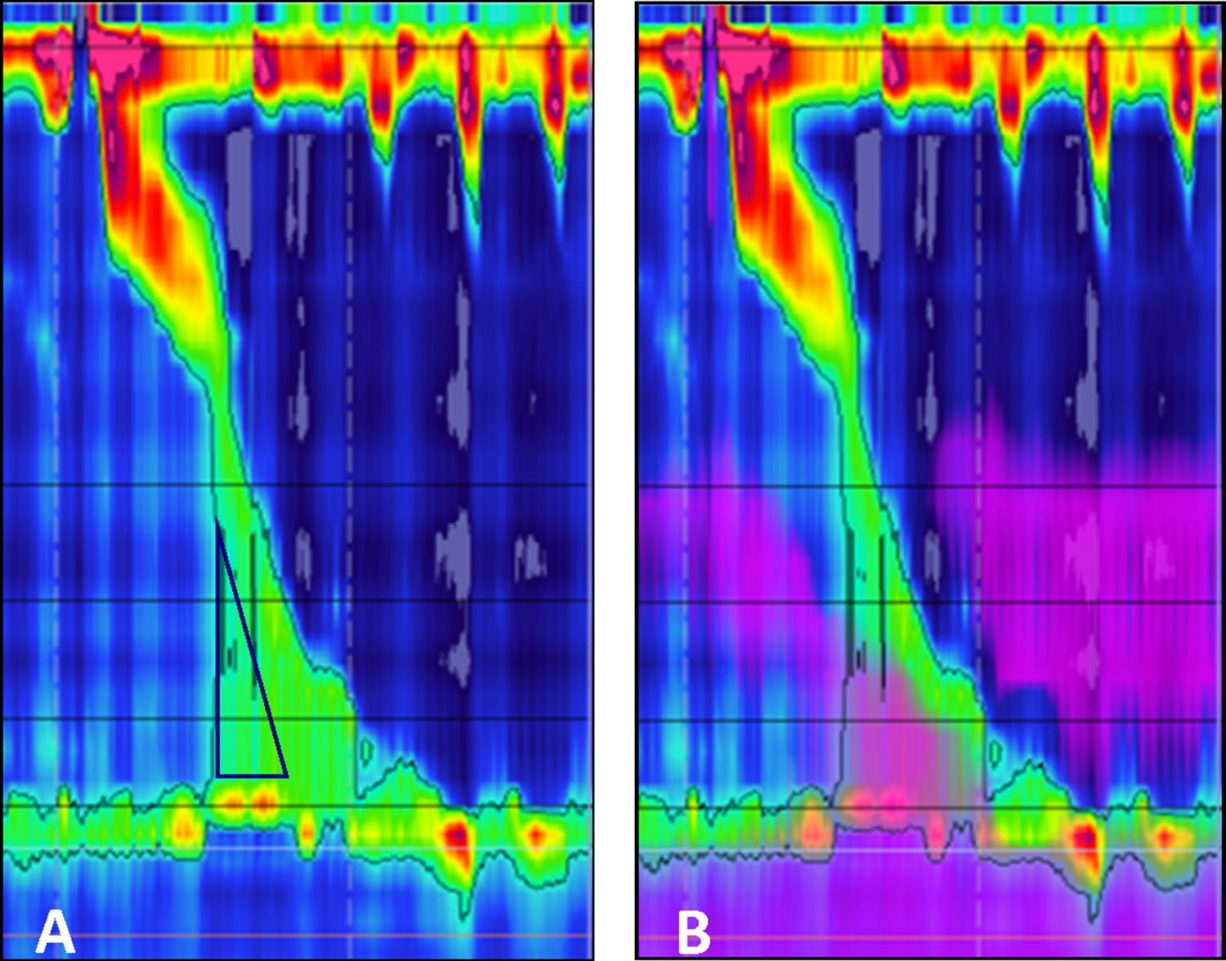
**A** HRM topographic plot of a patient with dysphagia after MSA. Elevated iBP can be visually identified (black triangle) as an area with increased pressure proximal to the magnetically augmented LES and preceding the esophageal contraction, lack of adequate deglutitive GEJ relaxation is also apparent. **B** Same HRM plot with superimposed impedance tracing showing lack of bolus clearance

Quader and colleagues posed the question of whether iBP could predict the presence of structural obstructive processes despite a normal IRP on HRM [23]. In this study of patients with esophageal dysphagia and normal IRP, the authors found that iBP may be significantly elevated in patients with obstructive processes compared to those without obstructive processes as evidenced on upper endoscopy. With this in mind, iBP may provide a more complete reflection of outflow resistance especially during the evaluation of dysphagia. While we only studied patients without dysphagia, it is important to highlight the fact that patients in the previous studies experienced dysphagia despite normal LES relaxation values [23]. The same phenomenon also may explain why patients with a normal IRP post-MSA may experience dysphagia and highlights the importance of iBP as a manometric metric. Unfortunately, the software tools embedded in the previous versions of the ManoView analysis software that enabled automated measurement of iBP is excluded in the latest version of the software. Elevated iBP can now only be appreciated visually on the spatiotemporal topography plots (Fig. 5) and measurement of the iBP requires the use of other software tools in the area above the LES to estimate the iBP.

Dysphagia remains the main concern during the preoperative workup and postoperative management of patients undergoing MSA. From the mechanical standpoint, dysphagia following MSA occurs when the contractile force of the distal esophagus attempts to overcome the resistance at the EGJ. Vagal afferent input is generated in this process, is highly variable among patients, and can be experienced as dysphagia even in the absence of obstruction on contrast or endoscopic examinations. There are multiple components to the resistive force at the EGJ, including the magnetic forces between beads, the native LES pressure, tissue compliance of the EGJ, and the fibrotic capsule which later forms around the device [17]. The attractive force between beads of a closed device is approximately 40g, slightly below that of common household magnets. The attractive force decreases to approximately 7g at maximal expansion of the LINX device [24]. Biomechanical in vitro experiments have shown that these forces are consistent across different magnetic ring sizes [25]. Interestingly, we also found that outflow resistance measured by iBP that is imposed by smaller size devices (≤ 14 beads) was not significantly different from those imposed by larger size devices (≥ 15 beads). This finding emphasizes that intra-operative device sizing should focus on caliber and position, rather than be used as a technique to modulate the degree of resistance.

LINX device provides magnetic attraction in the form of an expansible ring in the dynamic area of the EGJ. We found that post-MSA iBP was correlated with IRP and not resting pressure. This observation highlights the ideal design of the LINX that provides LES augmentation while not interfering with normal physiology. Magnetic forces are minimal during the passage of the bolus, and therefore, the resistance against bolus is correlated with pressure during LES relaxation, a physiologic vagally mediated process. In addition, decrease in magnetic force during esophageal displacement leads to less resistance with the larger the food bolus [26]. This is in contrast to the tissue reinforcement during fundoplication. Previous studies have shown increased resistance with progressively larger food boluses after Nissen fundoplication [27].

Native LES function and gastroesophageal junction compliance are other factors with potential impact on the EGJ resistance after MSA. Native LES function in patients with MSA is a factor which is yet to be fully understood and differentiating the magnetic ring from native LES pressure is not entirely feasible at least with HRM. Our study showed a strong correlation between post-MSA iBP and preoperative resting pressure. The EGJ compliance is a highly variable factor based on the severity and duration of GERD, as well as the extent of surgical dissection and the degree of fibrotic changes around the EGJ. Histological analysis of GERD patients has demonstrated that repetitive inflammation can lead to forming type-III collagen, fibrosis, and eventual structuring [28]. Tissue compliance of the gastroesophageal junction can be quite variable depending on the degree of preoperative GERD-related injury and postoperative inflammatory process. On subsequent surgical exploration, including explanations, a fibrosis capsule is typically encountered, encasing the MSA device. Capsular restriction may occur to varying degrees, depending on inflammation, connective tissue formation, and postoperative diet protocol [17].

**Fig. 6:**
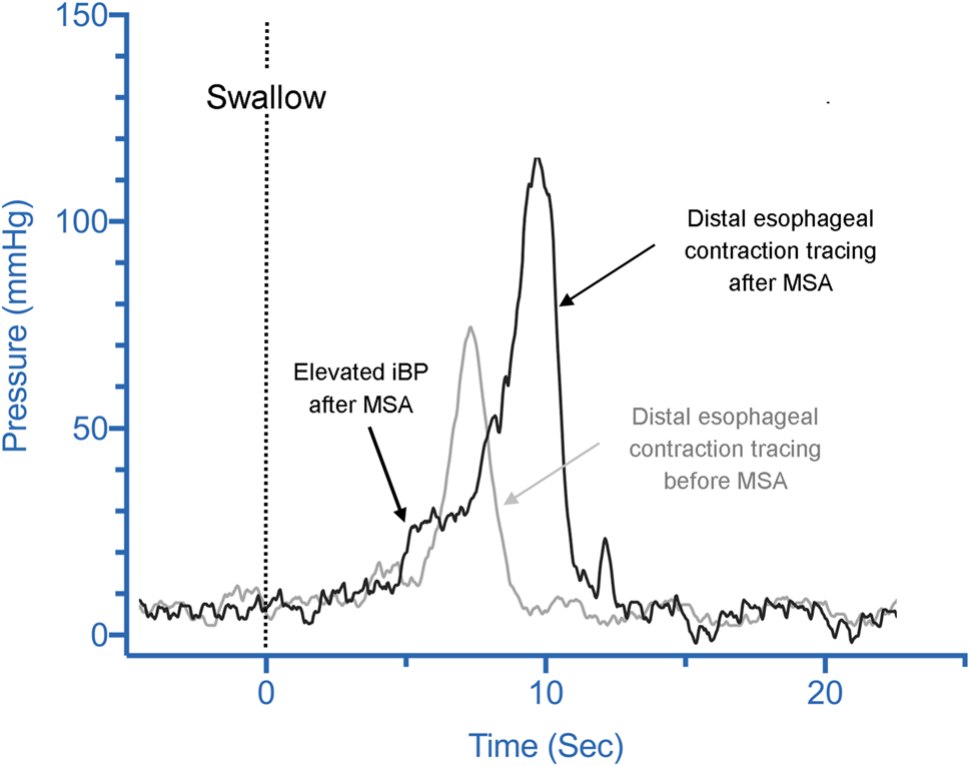
Distal esophageal contraction line tracing in a patient before (gray tracing) and after (black tracing) MSA. In these tracings iBP is recognized as a rise in pressure (ramp) to a plateau above the esophageal baseline just preceding the upstroke of esophageal contraction wave. This ramp pressure is significantly higher after MSA; note the higher distal esophageal contraction amplitude after MSA as a compensatory mechanism of the esophagus to overcome the outflow resistance of the magnetically augmented LES

Clinically, some resistive components can be distinguished by the chronology by which dysphagia occurs. Patients with early dysphagia, in the days following surgery, likely do not generate sufficient distal contractile force to overcome the magnetic forces between beads. Studies have noted significant increases in esophageal amplitude and DCI on HRM obtained a few months after MSA. It is reasonable to assume this compensatory increase in contractility can eventually overcome the resistance of the magnetically augmented sphincter (Fig. 6). Our finding of a direct correlation between post-MSA iBP and DCI supports this hypothesis. Although we found correlation between the degree of outflow resistance and incomplete bolus clearance, the compensatory increase in contractility resulted in maintaining the coordination of contractions evidenced by no change in % failed peristalsis and % bolus clearance when compared to baseline preoperative measurement. It is usually assumed that the majority of patients with adequate esophageal bolus clearance are free from dysphagia symptoms. In our study we found a significant correlation between iBP and incomplete bolus clearance on HRM. This finding is analogous to another recent study of non-MSA patients, which suggested that elevated mean iBP strongly correlates to liquid retention on timed barium esophagram [29]. Dysphagia, which onsets weeks to months following MSA, may be more suspicious for fibrotic capsular restriction, after fibrosis and collagen remodeling have occurred. If dysphagia persists, any compensatory increase in contractility is likely outweighed by outflow resistance.

We acknowledge that a limitation of our study is the lack of a control group of patients with dysphagia. This study was designed to quantify the normal outflow resistance of the EGJ after MSA in patients free of clinically significant postoperative dysphagia and to correlate this outflow resistance with manometric characteristics, bolus clearance, and the size of device. Therefore, our study population consisted of only those with no dysphagia after surgery. Our proposed threshold for normal outflow resistance needs to be tested in future studies that include patients with dysphagia.

## Conclusion

The EGJ outflow resistance measured by iBP is increased after MSA. The upper limit of normal for iBP was 30 mmHg in this cohort of patients who were free of dysphagia after MSA. Esophageal contraction amplitude in the distal esophagus must exceed this threshold in order to maintain antegrade bolus movement across the EGJ. The observed increase in iBP in this study is higher than values reported for laparoscopic Nissen fundoplication in the literature, emphasizing that there is a need to develop novel manometric criteria when selecting patients for MSA.
